# Towards a More Efficient Training Process in High-Level Female Volleyball From a Match Analysis Intervention Program Based on the Constraint-Led Approach: The Voice of the Players

**DOI:** 10.3389/fpsyg.2021.645536

**Published:** 2021-03-03

**Authors:** Carmen Fernández-Echeverría, Isabel Mesquita, Jara González-Silva, M. Perla Moreno

**Affiliations:** ^1^Department of Didactics of Musical, Plastic and Body Expression, Faculty of Sport Sciences, University of Extremadura, Badajoz, Spain; ^2^Faculty of Sport, University of Porto, Porto, Portugal; ^3^Faculty of Education Sciences, Psychology and Sports Sciences, University of Huelva, Huelva, Spain; ^4^Faculty of Sport Science, University of Granada, Granada, Spain

**Keywords:** athletes' perceptions, teaching-learning process, training, match analysis, volleyball

## Abstract

The aim of the research was to know the perception of high-level volleyball players of the changes produced (in relation to the previous season) in the efficiency of the training process, after a match analysis intervention program based on the Constraint-led Approach (CLA). The sample consisted of 11 players from a women's volleyball team. The protocol of the intervention program consisted of providing objective, contextualised and systematic information to the coach (adapted to his needs) that would allow understanding the different real game contexts. We used semi-structured interviews to assess players' perceptions. The athletes perceived changes in training, both in their preparation and development, specifically in greater involvement and organisation in preparing the training; in an increase in the specificity and suitability of training tasks according to individual needs; in the representativeness of the restrictions of the game; in a more tactical approach; in the variability of task and in the accountability to achieve the objective proposed. In addition, in the preparation and development for competition, the players detected more game planning; a deeper analysis of the opponents; an objective selection of the most relevant data, an increase in the depth of match analysis and the inclusion of the weekly meeting with the use of video compared to the previous season. These results expose the benefits of coaches incorporate programmes to obtain objective information about the game in their training process.

## Introduction

Performance analysis in sport can be done from different points of view (biomechanical, physiological, psychological, etc.) (Trecroci et al., 2018; Araújo et al., 2019; Formenti et al., 2019), among which we highlight performance analysis through match analysis as a fundamental tool to obtain information for the process of preparing for team sports (Butterworth et al., 2013; Filetti et al., 2017). As such, it has been introduced into the work routines of many teams (Palao and Hernández-Hernández, 2014). This information includes objective data that enables coaches to interpret the real context in which they are attempting to improve performance (Hughes and Bartlett, 2008). Match analysis has evolved over time. Initially, these analyses were conducted with the aim to obtain a general overview of competitions, but more recently they have been used to evaluate different aspects of the game and to be able to obtain the keys that guide our athletes to success. As a result, they have become more meticulous in their attention to the various game and situational variables (Fernández-Echeverria et al., 2017). Due to recent technological advances, there are now multiple statistical programmes that facilitate the analysis of the competition (Palao and Hernández-Hernández, 2014).

While some sport teams have pre-established key performance indicators, which are constantly evaluated through match analysis (Wright et al., 2013), it is necessary that these indicators can be modified according to the needs of the coach, depending on the match or the phase of a competition (Fernández-Echeverria et al., 2017). Therefore, if match analyses are not performed by the coach, and are instead conducted by an assistant coach or scout, it is important (a) for communication with the coach, (b) to identify the main features of the game (Wright et al., 2013); and (c) to select the important information for the team at each moment (Sarmento et al., 2015; Gesbert et al., 2016).

Several studies have shown that coaches consider match analysis to be important, easy to use, and useful for establishing objectives and planning the teaching-learning process of sport (Groom et al., 2011; Wright et al., 2012, 2014; Lago-Peñas and Gómez-López, 2014; Lupo and Tessitore, 2016; Kraak et al., 2018), evaluating results (Palao and Hernández-Hernández, 2014), and influencing the style and tactics of a game (Painczyk et al., 2017; Martin et al., 2018). This is because the information obtained from match analysis can help coaches to determine the key elements to develop in training (Wright et al., 2012; Renshaw et al., 2019; Loo et al., 2020). Understanding these key elements allows coaches to design more ecological and representative tasks (Renshaw et al., 2010; Chow, 2013; Woods et al., 2019) that give athletes a greater ability to adapt (Orth et al., 2019; Ramos et al., 2020a) to new circumstances and meet their needs (Greenwood et al., 2012). This adaptation process of the athlete to the environment has generated great interest in the scientific community, from which a theoretical perspective emerges, the Constraint-led Approach (CLA). The CLA understands that the athlete is in constant interaction with different conditioning factors that affect the appearance of behaviours creative and adaptive game-related (Davids et al., 2006). For this reason, CLA focuses on the manipulation of constraints during sports performance with the aim of facilitating the action-perception coupling of our athletes (Renshaw et al., 2016).

The information obtained from match analysis is not only used by coaches to plan the teaching-learning process or to design tasks of sport training. It is also transmitted to athletes (Wright et al., 2016). Currently, because it is vital to involve players in the pursuit of sports performance (Bampouras et al., 2012; Middlemas et al., 2018), high-level sports teams typically incorporate meetings into their training schedules in order to present information to their players (Groom and Cushion, 2004; Mesquita et al., 2005).

Players can be presented with information of various different forms, which can refer to personal or team performances, and can include statistical data and/or video clips (Cushion et al., 2006; O'Donoghue, 2006; Groom et al., 2011; Fernández-Echeverria et al., 2017). Moreover, this information can refer to the team itself and to rival teams (Painczyk et al., 2017). Analyses relevant to the team itself can provide information about the positive aspects of performance, in order to reinforce them, and the negative aspects of performance, to work and reduce them in training (O'Donoghue, 2006; Jenkins et al., 2007; Francis and Jones, 2014). Other analyses offer information on the strengths and weaknesses of rivals (Sarmento et al., 2015). This for the preparation of future matches is very useful (Groom and Cushion, 2004; Kraak et al., 2018).

Although several past studies showed the importance and usefulness of match analysis for sports coaches (Butterworth et al., 2012; Painczyk et al., 2017; Kraak et al., 2018), it remains unclear how the information obtained via match analysis, based on the Constraint-led Approach, can influence the teaching-learning process, specifically in the preparation and development of training and competition. One approach to addressing this research question is to ask athletes. Indeed, very few studies have been concerned with understanding the Athletes' opinions of the use of match analysis and its usefulness for training (Francis and Jones, 2014; Wright et al., 2016) and the changes this can provoke. Consequently, the objective of the study was to know the perception of high-level volleyball players of the changes produced (in relation to the previous season) in the efficiency of the training process after a match analysis intervention program, based on the Constraint-led Approach. The hypothesis of this study is that “high-level volleyball players will perceive changes in the efficiency of the training process (in the preparation and development of training and competition) after a match analysis intervention program, based on the Constraint-led Approach.”

## Materials and Methods

### Sample

Players from a women's volleyball team (*N* = 11) who competed at a high level formed the study sample. The age of the players was between 18 and 32 years old (*M* = 26.09, *SD* = 4.45). All the players had basic previous experience in performance analysis, which allowed the application of the program without any problem. The team were trained 5 days a week, specifically 9 h a week on the court and with a weekly competition game (Saturdays).

All participants were guaranteed confidentiality and anonymity throughout the process. The investigation was carried out under the recommendations of the Declaration of Helsinki. The participants were informed of the study and signed a consent form. It was in accordance with Spanish guidelines for scientific research in human beings.

### Protocol for the Match-Analysis Intervention Programme Based on Constraint-Led Approach

The protocol of the intervention programme consisted of the provision of objective, contextualised and systematic information. Specifically, the information given to the coach (adapted to his needs), pertaining to the competitive performances of the players of the study team and their opponents, was obtained via match analysis (Fernandez-Echeverria et al., 2019).

The programme was applied by the study's principal investigator who was also the assistant coach. This role in the team was suitable for applying the intervention programme due to the trust between the head coach and the assistant coach. In addition, we emphasise that the reports were completed based on the coach's needs (Wright et al., 2013). For the intervention programme, the assistant coach made scout reports (relating to the performances of the study team and their opponent) for each match and provided them to the coach. This occurred for each game during the period of a full competitive season. The season lasted 6 months. This season was composed of two phases of 3 months each (first and second matches).

The intervention programme consisted of several phases for each game. During phase one, or the diagnosis and game analysis phase, data was collected by video recording the match from the back of the court (this guarantees an optimal line of sight) and this was later used to conduct a match analysis (study of the own team and the rival). During phase two, or the elaboration and provision of information phase based on the pedagogical principles of the CLA, the information obtained from the match analysis was collated in two scout reports (one related to the performance of the study team and the other relating to the performance of the opponent). These two reports were then presented at different times: the report related to the study team was delivered to the coach at the beginning of the week, and the opponent report was delivered midweek.

To facilitate the coach's understanding of the data, the reports contain only the most important data (Hughes and Franks, 2004). The reports concerning performance of the study team focused on several types of contextualised information: (a) Specific game situations of different phases of the game (KI attack phase and K2 defence phase) (Beal, 1989) that required correction or improvement. Specifically, the objective of the attack phase is to build an attack to obtain the point (setting, reception, attack, and attack coverage) and the objective of the defence phase is to defend the attack to build a counterattack that allows to obtain the point (includes serve, block, defence, setting, counterattack, and counterattack coverage). (b) Individualised technical-tactical information for each player, including the positive aspects to highlight and the negative aspects that need to be reduced or corrected (O'Donoghue, 2006; Jenkins et al., 2007). The information presented to the coach concerning the study team was obtained by analysing multiple variables (game and situational variables) relevant to both the team and its individual players (see Table 1).

**Table 1:** Aspects of the game considered for own team reports.

***Game variables:*** • **Serve:** serve type, serve zone, serve efficacy, etc. (Fernandez-Echeverria et al., 2015) • **Receptión:** recepción zone, reception type, reception efficacy, etc. (Afonso et al., 2012) • **Set:** setting technique, set's area, tempo of a set, setting efficacy, etc. (González-Silva et al., 2016) • **Attack:** attack type, attack tempo, attack efficacy, etc. (Castro et al., 2011) • **Coverage:** coverage zone, coverage efficacy, etc. (Hileno and Buscà, 2016) • **Block:** block type, block eficacy, etc. (Marcelino et al., 2009)	***Situational variables:*** • **Set** (García-Hermoso et al., 2013) • **Rotation** (Ðurković et al., 2008) • **Game Complex (K1/K2)** (Castro and Mesquita, 2010) • **opposition quality** (Marcelino et al., 2012) • **Score** (Marcelino et al., 2011) • Match period (Marcelino et al., 2012)

The opponent reports focused on contextualised information about the characteristics of the opponent's game system, players, game tendencies, and game model. In addition, these reports took into account other types of information including the possible player changes during the course of the game, the characteristics of the coach, the characteristics of the court, the public, etc. (see Table 2). Because understanding the weaknesses and strengths of an opponent can help guide a team's strategic plan (Groom and Cushion, 2004), the above information was collected to help the study team prepare for future encounters with their opponents.

**Table 2:** Aspects of the game considered for the opponent team reports.

**Characteristics of the game system** (Mesquita et al., 2013): • Reception • Attack • Defence (1st and 2nd line) • Coverage	**Characteristics of the players** (Quiroga et al., 2010): • The strongest and weakest players at reception and their characteristics • The strongest and weakest players at attack and their characteristics • The strongest and weakest players at blocking and their characteristics • The strongest and weakest players at defence and their characteristics
**Game tendencies for rotations** (Ðurković et al., 2008): • Serve • Distribution of attack • Direction of attack	**Rival game model** (Fernández del Valle et al., 2009): • Technical-tactical intentions of the opponent in the final moments of set and match • Initial rotations in serve possession and in reception
**Other relevant information** (Campos et al., 2014): Player substitution, coach characteristics, court characteristics, nature of public, etc.	

Finally, in phase three, or the phase of providing information to players, the report was delivered to the coach, and the coach then transmitted the information to the team through reports and video clips (see Table 3). Video clips related to the positive and negative game actions performed by the players, as well as the opponent's game in the attack and defence phases. Information was presented to players in two separate meetings each week. Specifically, at a post-match meeting held on Monday and a pre-match meeting held on Thursday (Middlemas et al., 2018). The purpose of the post-match meeting was to analyze the scout reports related to the team itself and to visualise, using video clips, the negative and positive aspects of their game. The aim of the pre-match meeting was to evaluate the scout reports related to the opponent team and to visualise, using video clips, different game situations of the opponent in the defence phase (K2) and attack phase (K1). All pre-match and post-match meetings were held with the full team, before starting the training session. The same pattern has been followed in the transmission of information with small changes throughout the season according to the needs of the team and the coach. In addition, in both meetings, an emphasis was given to transmitting information to players clearly and to focus on the most relevant aspects.

**Table 3:** Weekly summary of activities providing information.

**Weekdays**	**Days of training sessions and match**	**Activities**
Monday	Training session	Post-match meeting with the full team (before the training session). Providing information to players about the team's performance and video clips (post-match report).
Tuesday	Training session	Analysis and study of the opponent's game (next match) carried out by the scout. Delivery of the pre-match scout report to the coach
Wednesday	Day of rest	
Thursday	Training session	Pre-match meeting with the full team. Providing information to players of the opponent's study and video clips (pre-match report).
Friday	Training session	
Saturday	Match day	Video recording of the match/statistics.
Sunday	Day of rest	Analysis and study on the performance of the team itself carried out by the scout. Delivery of the post-match scout report to the coach.

In addition, the intervention programme (based on delivering objective, contextualised, and systematic information tailored to the needs of a high-level volleyball coach) influences a number of features of the preparation and development of training and for competition (see Table 4).

**Table 4:** Features of the intervention programme.

**Preparation and development of training**	Increases the involvement of the coach in the training process
	Increases tactical work
	Increases contextualisation of training tasks
	Increases the specificity of training tasks
	Increases variability of training tasks
	Involves and increases responsibility of players for achieving the proposed objectives
**Preparation and development for competition**	Increases the clarity of transmitted information
	Increases the depth and specificity of game analysis of the team and their opponents
	Makes the game analysis of the team and their opponents more systematic
	Inclusions of the weekly meeting with the use of video clips to complement game analysis of the team and their opponents.

### Data Collection and Instrument

Data was collected using a semi-structured interview technique. This technique was applied with the aim to understand players' perceptions of the changes produced in the teaching-learning process of hight level sport. Eleven players who were part of the team during the intervention season and season prior (season in which not the match analysis of the own team and the rival provided to the coach was carried out based on Constraint-led Approach) to it were interviewed so that they could answer comparative questions. This was due to the fact that one of the players was not part of the team last season and could not answer the questions aimed at comparing the two seasons. Consequently, 11 of the 12 players who were part of the team were interviewed at the end of the competitive season involving the intervention programme. This specific moment was chosen so that the athletes could make a full assessment of any changes across the season, in relation to the prior seasons, after the intervention programme. The interview consisted of the questions presented: Do you perceive any change or modification in the preparation and development of training with respect to the previous season? If yes, indicate what these are. Do you perceive any change or modification in the preparation and development for competition with respect to the previous season? If yes, indicate what these are.

The interviews were recorded with a digital recorder (Olympus VN-712PC) in a quiet room in the pavilion where the team trained. The average duration of each of the interviews with the players of the team was 12.32 min. The contents of the interviews were transcribed verbatim in order to obtain an accurate and complete record of the data collected. During the development of the interviews, the main researcher acted as an active listener (Smith and Sparkes, 2005).

### Data Analysis

For data analysis, we carried out a thematic analysis following the process suggested by Charmaz (2014). Concretely, various phases were carried out. The first phase was based on a repeated reading of the transcripts was carried out and an open data coding. All this, with the aim of segmenting the data and listing a series of subcategories and categories. The second phase was based on axial coding to subsequently philtre the subcategories and categories of the first phase (Table 4) (guided by specialists in qualitative research and volleyball). The third phase was based on selective coding. Specifically, the researchers selected two main dimensions (preparation and development of training, and preparation and development for competition) and then related all the other subcategories and categories with them (Charmaz, 2006, 2014). To contextualise the results, a review of the current literature was carried (Holt and Dunn, 2004). Finally, we focus on refining the relationships between the categories (report between the first author and co-authors).

### Trustworthiness

To optimise the credibility of the research, we carried out several steps suggested by the literature (Biddle et al., 2001). First, the principal investigator (interviewer) carried out a training period supervised by an investigator with experience in qualitative analysis and in semi-structured interviews (Patton, 1990). Second, several meetings were held by a group of specialists in qualitative methodology and volleyball (three volleyball specialists with a level III coaching qualification) to establish the category system (Meyer and Wenger, 1998). In addition, the players were asked to review the transcript of the interviews for verification, which will allow adding, deleting or reworking any information that they consider did not accurately reflect the information previously provided (Corbin and Strauss, 2015). All these steps are necessary for the credibility of the data in qualitative research (Silverman, 2000).

## Results

The results indicated that the athletes perceived that their coach was more efficient, than the previous season, in the preparation and development of training and competition from the use of Match analysis information based on CLA.

Specially, the players perceived changes in training preparation and development, compared to the previous season: (1) in greater involvement and organisation in preparing the training; (2) in an increase in the specificity of training tasks; (3) in an increase in the suitability of training tasks according to individual training needs; (4) in the representativeness of the restrictions of the game in the training task (by the use of a more tactical approach); (5) in a more variability of tasks; (6) in the accountability to achieve the objective proposed by the coach and complete the tasks.

In addition, the players perceived in the preparation and development for competition: (1) changes in the match analysis of opponents (with a more specific, deep and clear match analysis that provides great information to analyze and that helps athletes decide what to do during the game); (2) changes in the match analysis of own team (with an objective selection of the most relevant data, in a clear and concise way, a greater depth of analysis due to the incorporation of positive and negative aspects and the inclusion of a weekly meeting with the use of video compared to the previous season).

### Preparation and Development of Training Compared to the Previous Season

Our results show that 81.81% of the team's athletes perceived changes in the preparation and development of training compared to the previous season. Specifically, 54.54% of the athletes referred to changes in the coach and 63.63% referred to changes in the training tasks (see Table 5).

**Table 5:** Descriptive analysis of the changes and modifications perceived by players in the preparation and development of training.

**Categories**	**Subcategories**	**Frequency**	**Percentage**	**Players**
Changes in the coach		6	54.54%	2/4/5/6/7/10
	Greater involvement of the coach in training preparation (work to be done written on paper and use of blackboard or notebook)	6	54.54%	2/4/5/6/7/10
Changes to the training tasks		7	63.63%	1/3/4/5/6/7/9
	Increase in tactical work (individual and collective)	3	27.27%	1/4/7
	Training tasks more contextualised (simulation of rival game)	4	36.36%	1/4/5/7
	Increased specificity of training tasks (tasks specific to roles and tasks designed to develop game situations that require improvement)	6	54.54%	1/4/5/6/7/9
	Greater variability of tasks (tasks with distinct variants and alternatives of action)	1	9,09%	3
	Introduction of control mechanisms so that athletes are responsible for completing the task–*accountability* (use of scoring in tasks)	2	18.18%	4/5
Total		9	81,81%	1/2/3/4/5/6/7/9/10

The results indicated that the coach was more involved and more organised in training preparation because of the intervention according to the players' perception (54.54%). The perception of players was that in the previous season training sessions were less prepared. In contrast, players indicated that the intervention programme changed the coach: the coach had prepared written training sessions and supported his explanations of exercises using a notebook and a blackboard.

We have observed more preparation in all senses, at all levels… the training tasks were much more prepared than in previous years. (Player 5)

I realised that the coach has prepared the training much more, was all the time with notes or the blackboard. This year, it was noticed that the coach was studying a lot, he always knew what he wanted to train and he had everything in his head. However, last year in training they improvised more, I was not as prepared as this year. (Player 6)

Concerning the perceived changes to training tasks, our results show that most of the players referenced an increase in the specificity of training tasks (54.54%). The players indicated that the coach had increased the use of tasks that were specific to game roles during the intervention season and had incorporated new materials, such as a precision hoop to help develop the pass of the setters.

In addition, this year we have done a minimum of two specific tasks per week for setters. Lately, with the precision hoop, which is very important because the previous year we did not. Carrying out specific training came in handy because accuracy is very important. (Player 5)

Moreover, there is an increase in the suitability of training tasks according to individual training needs. Specifically, there was an inclusion of tasks to work on specific game situations or aspects that the players needed to improve.

This year we trained very specifically, for example it was suggested that we were going to play against a specific team and they trained certain balls from a player specific to that team… (Player 7)

Another of the perceived changes was an increase in the representativeness of the game restrictions in training tasks that simulated the rival's game. This caused an increase in the contextualisation of the training tasks (36.36%).

…*we trained simulating the game of the other teams, in some parts of the training, as we tried to do the same thing that the rivals did against those we were going to face. I think that has been very good for us. (Player 1)*

In addition, our results indicate a more tactical approach through an increase of tasks focused on tactical work (27.27%), including tasks to develop individual and team tactics.

This year we have trained very tactically. (Player 7)

Perhaps we have trained more tactically to improve certain things individually. This is very good because we have improved many aspects of the game. (Player 1)

There was also a perceived increase in the control mechanisms used to encourage athletes to be responsable and accountable for reaching the goal proposed by the coach and completing tasks (18.18%). These included giving extra points if a goal was achieved or by subtracting points for any errors committed. In the previous season, the coach gave no importance to these issues.

Perhaps in this second part we have gotten more games with points, where the goal was to get them and if you made a mistake you subtracted. For example, if you missed the serve, they took more points. This came in handy because you put a little more pressure on the training, and you demand more than when it does not matter if you miss 10 serves or one. This will make you do better in matches… it's very good. (Player 5)

This year we have learned to think more, for example last year maybe you just started playing without thinking about so many questions and this year… everything had a goal. (Player 4)

Finally, the results show an increase in the variability of tasks compared to the previous season (9.09%), through a modification of the constraints that promote work under different game situations.

This year we have trained more different things compared to the previous year, we have trained with a greater variability of exercises. (Player 3)

### Preparation and Development for Competition Compared to the Previous Season

Our results show that 100% of the team's athletes perceived changes in the preparation and development for competition compared to the previous season. Specifically, 90.9% of the athletes referred to changes in the match analysis of opponents and 81.81% related to changes in the match analysis of the study team (see Table 6).

**Table 6:** Descriptive analysis of the changes perceived by the players in the preparation and development for competition.

**Categories**	**Subcategories**	**Frequency**	**Percentage**	**Players**
Analysis of opponents		10	90.9%	1/2/3/4/5/6/7/8/10/11
	Increase the clarity of the transmitted information (objective selection of the most relevant data in a clear and concise form)	4	36.36%	1/6/10/11
	Increase the depth of match analysis (detailed analysis of players and the game)	9	81.81%	2/3/4/5/6/7/8/10/11
	Increase the specificity of match analysis (analysis according to different situational variables)	8	72.72%	3/4/5/6/7/8/10/11
Analysis of own team		9	81.81%	1/2/3/4/5/7/8/9/11
	Game analysis is more systematic (weekly meeting and analysis)	3	27.27%	1/3/11
	Increased depth of match analysis (inclusion of positive and negative aspects of the game)	7	63.63%	1/2/3/4/5/9/11
	Incorporation of the weekly meeting with the use of video clips (phases of play and plays cut into clips)	5	45.45%	2/4/7/8/9
Total		11	100%	1/2/3/4/5/6/7/8/9/10/11

Concerning the changes perceived in the analysis of opponents, our results indicate that the players perceived deeper analysis of opponents (81.81%) to give players more information to analyze and decide what to do during the game. This is due to a detailed analysis of the player and match characteristics. Concretely, information is provided on the strongest and weakest players in the various game actions (reception, attack, blocking, and defence) and their characteristics.

This year an in-depth analysis was made, focusing more on the players individually. However, before comments were made only if one stood out, and now with the statistical analysis you get many details, I think it is better. In addition, this is how the week is set according to the team that we are going to face, depending on its characteristics. I think it has developed much better than last year. (Player 3)

In addition, players indicated a greater specificity of analysis of the opponents' game (72.72%). Specifically, the players indicate a more detailed analysis that considered situational aspects such as game rotations. We highlight that service information, attack distribution, and attack direction were presented at each game rotation.

Totally, last year was very different from this one, we made it more specific, last year we watched videos and talked about people in a general way, but now with the theme of rotations, we analyze each player, in each rotation, and so we know more about them. (Player 11)

Last year there were no reports, there was nothing specific. Now they give you the reports and indicate specific aspects of the opponent such as this player attacks in this way… (Payer 5)

The players also highlighted that with this programme there was an increase in the clarity of information transmitted to them (36.36%). Specifically, the players perceived that they were shown an objective selection of the most relevant data, in a clear and concise manner, while in the previous season they observed game videos that were too long and thus caused a loss of attention.

Yes, this year there have been many changes, the reinforcement of all the data that you gave us when it comes to studying the players. Now everything is much more clear than other years. (Player 1)

For example, now we see a team serve and its reception in all rotations and this is much better than last year. Before, the coach showed us a whole set and it was too long to watch. The girls started talking about other things like the player's hair, we were not focused on what we had to look at. However, this year is more clear, if you put each rotation, it is clearer, because half an hour of video is a lot. This has also changed from last year to this one. (Player 6)

Concerning the evaluation of the team's own game, most of the players referred to a greater depth of analysis due to the incorporation of positive and negative aspects (63.63%) in pre-match meetings, which were not held in the previous season.

In the evaluation of the game, the comments on the errors and successes is good. Last year we did not do it, we commented a little, but we did not have a meeting to discuss the mistakes and the successes. (Player 3)

The players also highlighted the incorporation of video to complement the analysis of their teams' game in the intervention season (45.45%). They emphasised the inclusion of detailed images of different aspects of the game that helped players to be aware of the positive aspects to be maintain and the negative aspects to be corrected or improved.

Last year we used videos, for example to see the next team, but to see us we did not use anything. This is a big change because we have improved a lot in the analysis of our own team, both in terms of the use of statistics and in the videos you showed us, to be aware of the failures and improvements. (Player 4)

Last year there was also an assessment of the game and the coach spoke to us. However, this year we have videos of different moments of the game and this, the images, count a lot, with this we see many of the aspects of the game that we do wrong. (Player 8)

The inclusion of a weekly meeting was definitely important to give the players the opportunity to reflect and make a systematic analysis of the team's own game compared to the previous season (27.27%).

Last year we analysed the games we had had a little bit after, we did not meet every Monday, as we do this year…the meetings were very important to assess what we had done well or failed in the game. (Player 1)

## Discussion

The aim of the research was to know the perception of high-level volleyball players of the changes produced (in relation to the previous season) in the efficiency of the training process, after a match analysis intervention program, based on the CLA.

The results of the study indicated that after the application of the intervention programme, athletes perceived changes in training and competition both in preparation and development. In relation to perceived changes in the preparation and development of training, the players perceive that the coach is more involved and organised in the preparation of the training. The players referred to a greater involvement of the coach in the preparation of training as evident by the use of prepared written work plans, the absence of a need to improvise due to lack of preparation, and continually using notes or the blackboard to support explanations of tasks. In the season prior to the intervention, training session were reported to be less prepared. Studies that evaluate players' perceptions of the possible changes in training that can result from using match analysis are scarce. Nonetheless, our findings align with those that demonstrate match analysis facilitates an understanding of the weaknesses and strengths of a team and its rivals, and that they help coaches to prepare training and to establish objectives (Silva et al., 2011; Wright et al., 2012; Sarmento et al., 2015). Further, it is likely that when coaches are clear about their goals and the means to achieve them they may be more motivated and thus present a greater engagement in daily work.

Another of the changes perceived by the athletes regarding the preparation and development of training was in the design of training tasks. Specifically, the players detected an increase in the specificity of training tasks (increased use of tasks specific to game roles and development of specific game situations), in the suitability of the training tasks according to the individual training needs, in the representativeness of the restrictions of the game in the training tasks, in the more tactical approach through a increase of tasks focused on tactical work, in the variability of tasks (increased use of tasks with different variants and alternatives of action) and in the accountability to achieve the objective proposed by the coach and complete the tasks, compared to the season prior to the intervention.

It is possible that these changes were due to objective knowledge, obtained from match analysis, being transmitted to the coach and thus enabling them to understand the key aspects of the game to work (O'Donoghue, 2006; Laird and Waters, 2008; Wright et al., 2012; Loo et al., 2020). Concretely, with such information it is possible to identify the constraints that must be manipulated in the design of tasks in order to create representative work environments (Renshaw et al., 2010; Chow, 2013), which meet the needs of athletes (Greenwood et al., 2012) and that guide the exploration of effective solutions in challenging environments (Roberts et al., 2019) with the aim of improving their performance (Hanin et al., 2016). In this sense, there are several studies that recently expose the importance of this ecological perspective for coaches in the advancement of training design (Ramos et al., 2020b; Woods et al., 2021).

The obtained tactical information about the team's opponents, as well as the information about the performance of the team itself, will increase the coach's knowledge of the game (Nash and Collins, 2006). This helps the coach to adjust their game model, determine the tasks with which to work during the week (Silva et al., 2011; Lago-Peñas and Gómez-López, 2014; Lupo and Tessitore, 2016), and to increase the possible variants of each exercise (Da Matta, 2015), all of which were referred to by the athletes interviewed in the current study.

In addition, objective information concerning a team and their opponents helps the coach to understand real-game scenarios, which in turn will allow them to design training tasks that simulate those contexts (Da Matta, 2015; Sarmento et al., 2015). As such, training tasks will be more representative of the game (Pinder et al., 2011, Vickery and Nichol, 2020) and allowing players to make decisions and solve problems that arrive during the competition (Davids et al., 2008). Moreover, in the pursuit of performance and the achievement of objectives, the accountability of tasks will be increased, that is, the way in which the coach ensures that athletes are responsible for completing the task and reaching the proposed objectives (Silverman et al., 1995).

Concerning the players' perceptions of changes in the preparation and development for competition, our results showed changes to the quality of game analyses. This applied to the analyses of the study team and their opponents. In particular, the players detected more game planning, a deeper (the strongest and weakest players in the various game actions and their characteristics) and specificity (service information, attack distribution and attack direction were presented at each game rotation) analysis of the opponents to give them more information to analyze and decide. In addition, an objective selection of the most relevant data was detected, in a clear and concise way, a greater depth of analysis due to the incorporation of positive and negative aspects and the inclusion of the weekly meeting with the use of video. All of this was important to give players a chance to reflect and make a more systematic analysis of the team's game compared to the previous season.

There are numerous studies demonstrating the importance of performing an analysis of opponents (Wright et al., 2012, 2013; Palao and Hernández-Hernández, 2014). Although research focused on players' perceptions of this topic is scarce, some studies have shown that prior knowledge of a rival helps athletes to be more prepared for competition and increases the chance of winning future encounters (Francis and Jones, 2014). An analysis of an opponent's game allows coaches to define the game strategies needed to overcome the opponent, helps define their strengths and weaknesses, guides the design of training tasks, and helps determine the best strategic plan (Silva et al., 2011). Although the relevance given by coaches to the information extracted from match analysis has been highlighted (Wright et al., 2012; Palao and Hernández-Hernández, 2014), it is also necessary that this information is transmitted efficiently and systematically to players (Wright et al., 2012; Fernández-Echeverria et al., 2017). It is, therefore, necessary to implement the use of match analysis in sports teams and to analyze the most efficient mechanisms for transmitting this information considering athletes' opinions.

Another factor indicated by the players was their perception that the game analysis of their team had changed. Specifically, players indicated that after the intervention programme there was an increase in the depth of the analysis (due to focus on both negative and positive aspects of performance) and the inclusion of the weekly meeting with the use of video (emphasising the inclusion of detailed images of aspects of the game that help the players to be aware of their failures and their successes). In addition, the players perceived that their game analysis was more systematic due to the inclusion of a weekly meeting in the training process for analysing team performance.

In a similar vein, several studies have shown the importance of analysing the performance of one's own team in addition to the performance of rivals. For example, a study by Silva et al. (2011) has shown that game analysis of a team helps; (a) consolidate their way of playing, (b) identify the weaknesses and strengths of the team and individual players, (c) correct individual and team errors, and (d) design training tasks according to the needs of the team. It is, therefore, necessary that analyses of a team are conducted systematically and focused on an evaluation of positive aspects, in order to reinforce them, and negative aspects, in order to correct in training (O'Donoghue, 2006; Jenkins et al., 2007). A study by Francis and Jones (2014) highlights the importance given to match analysis by players as a means to understanding the aspects of the game that they have to change, in line with more current studies such as that of Woods et al. (2019). Moreover, the transmission of visual feedback to players can be enhanced if game analyses of a team are accompanied by images or videos (Groom et al., 2011; Nelson et al., 2011; Booroff et al., 2016). Such visualisations present different aspects of the game that athletes are often unable to remember (Groom and Cushion, 2004) and are considered important by players due to their need to evaluate and reflect on performance (Wright et al., 2016).

## Conclusions

In the present study, the athletes reported that they thought match analysis intervention program, based on the Constraint-led Approach caused positive changes in training and competition, both in preparation and development. Specifically, athletes perceived a greater participation and organisation in training preparation, more representative and contextualised task design, more tactical work, more mechanisms that allow to control that the athletes assume the responsibility for completing tasks (accountability), and increased specificity and variability of tasks. Additionally, the players reported improvement to the game analysis of opponents (with deeper and more specific analysis and increased clarity of transmitted analysis), and of their own team (with an increase of systematic analysis focused on both positive and negative factors and the inclusion of the weekly meeting with the use of video). Therefore, as recommendations for practise, we expose the importance of coaches incorporate programmes to obtain objective information about the game via match analysis, taking into account the ecology of the game. These programmes must be developed according to the needs of the team, with detailed analysis of both the team itself and their opponents. This information will allow coaches to identify the most relevant elements of the game and facilitate a representative design of tasks according to the reality of the team. In relation to the limitations of the research of our study, we expose that it has been carried out in a single high-level women's volleyball team, so we suggest to researchers that this type of field research work continue in the future in various sports teams of different gender, category, and sports modality.

## Data Availability Statement

The raw data supporting the conclusions of this article will be made available by the authors, without undue reservation.

## Ethics Statement

The studies involving human participants were reviewed and approved by University of Extremadura. The patients/participants provided their written informed consent to participate in this study.

## Author Contributions

CF-E, IM, and MM: conceptualisation and methodology. CF-E, IM, JG-S, and MM: formal analysis. CF-E and MM: investigation and conceived and designed the research. IM, JG-S, and MM: supervision and reviewed and discussed the results. CF-E: writing—review and editing, drafted the manuscript, and prepared figures. MM and IM: revised the manuscript. All authors have read and agreed to the published version of the manuscript.

## Conflict of Interest

The authors declare that the research was conducted in the absence of any commercial or financial relationships that could be construed as a potential conflict of interest.
